# Physicians’ expectations of the use of conversational agents in healthcare: a qualitative study

**DOI:** 10.1186/s12913-026-14321-8

**Published:** 2026-03-12

**Authors:** Maximilian Wutz, Sara Söling, Juliane Köberlein-Neu

**Affiliations:** Center for Health Economics and Health Services Research, Schumpeter School of Business and Economics, University of Wuppertal, Rainer-Gruenter-Str. 21, 42119 Wuppertal, Germany

**Keywords:** Conversational agents, Chatbots, Digital health, Artificial intelligence, Healthcare innovation, Implementation barriers, Physicians’ perspectives

## Abstract

**Background:**

Conversational agents (CAs) have become an emerging field of research in healthcare, driven by their potential to support medical workflows, improve efficiency and enhance access to health services. However, despite growing technological development, their actual implementation in healthcare remains limited. In particular, little is known about physicians’ expectations regarding the potential use of CAs in healthcare. This study therefore investigates physicians’ expectations toward CAs and identifies expected barriers and facilitating factors for a possible future implementation.

**Methods:**

We conducted semi-structured interviews with 16 physicians from various specialties and care settings (inpatient and outpatient) across Germany. Maximum variation sampling was applied to ensure diversity in gender, age, prior experience with CAs, and medical discipline. The interviewees were encouraged to share their expectations regarding the potential use of CAs, their preferred characteristics, and expected barriers and facilitators for future implementation. Interview data were analyzed based on the concept of the Framework Method.

**Results:**

Five main topics were identified regarding how physicians expect the potential use of CAs in healthcare. Only one of sixteen physicians reported practical experience with a medical CA, highlighting a critical gap between expected potential and real-world application. Physicians viewed CAs as promising tools for maintaining the quality of care in the face of increasing workloads and staff shortages. Expected benefits included time savings, efficiency gains, and improved patient access, particularly through administrative support, symptom assessment, and patient empowerment. At the same time, substantial expected barriers were reported, including legal uncertainties, lack of interoperability, data privacy concerns, and limited real-world experience. Physicians emphasized that the true potential of CAs becomes apparent only through practical application.

**Conclusion:**

This study provides insights into physicians’ expectations regarding the potential use of CAs in healthcare and key factors for future implementation. According to physicians, successful implementation requires consideration of technical performance, regulatory frameworks, integration into clinical workflows, and human factors such as acceptance, trust, and user competence. CAs should support decision making without undermining clinical judgment. Practical trials and pilot programs are essential to foster trust through transparency and real-world experience, providing an orientation for evidence-based implementation initiatives.

**Supplementary Information:**

The online version contains supplementary material available at 10.1186/s12913-026-14321-8.

## Background

The healthcare sector is undergoing a digital transformation, and artificial intelligence (AI) is expected to have a significant impact on future medical care [1]. One promising technology using AI is conversational agents (CAs), also known as chatbots. CAs are digital dialogue systems that enable people to have text-based, voice-based or non-verbal conversations with a computer or machine as a conversation partner via an interface [2]. The potential application areas of CAs in healthcare are diverse and include administrative support (such as appointment scheduling, documentation, and reminders), symptom assessment and triage, patient education and empowerment through evidence-based information, support for clinical decision-making and diagnostics, as well as behavioural and mental health interventions [3, 4].

Although the evidence base is still emerging, many studies point to the potential benefits of CAs for healthcare. They suggest that CAs can help improve access to healthcare services, support clinical decision-making, reduce the workload for healthcare professionals, and improve patient-centered care [3, 5]. Several studies also indicate that CAs can be valuable tools in specific contexts, such as diagnostic support or mental health interventions, and highlight potential efficiency gains in clinical processes [3, 5, 6]. Furthermore, studies emphasize that CAs can help reduce costs and increase the scalability of services, making them particularly attractive for healthcare systems with limited resources. However, the robustness of the evidence varies considerably between areas of application. Recent reviews emphasize that most studies highlight the potential of CAs, while objective evidence of effectiveness, efficiency, or cost savings remains limited and is rarely measured using robust study designs [3, 5]. Furthermore, the actual use of CAs in clinical care is still rare, and satisfaction among physicians is mixed [7].

Alongside these promising opportunities, studies show that the implementation and sustainable integration of these technologies into routine care poses considerable challenges. The most significant obstacles include insufficient user-friendliness, lack of interoperability with existing systems, uncertainties regarding data protection and liability, and limited user confidence in these technologies [3]. In addition, deficiencies in system design, the risk of overuse, and a potentially excessive dependence on CAs are discussed as critical issues [8]. Risks such as misdiagnosis, overdiagnosis, and a possible shift in responsibility are also highlighted as relevant challenges [4]. These aspects underscore the need to critically analyse both the advantages and limitations of CAs and to systematically incorporate existing barriers into future implementation strategies.

As a result, research into the implementation of CAs in healthcare has increased significantly in recent years. In particular, the factors influencing implementation outcomes and how potential users make sense of the application of CAs have become an important area of interest [7, 9, 10]. Studies in this area have shown that CAs are associated with poor implementation readiness among physicians and patients [11]. However, little is known about the perspectives of physicians [7, 12]. The main focus of research has been on patients as users [2, 3, 5].

This research gap regarding the perspective of physicians must be addressed, as the stakeholders in the use of CAs in healthcare include not only private users and patients, but also physicians. Their attitudes, beliefs and experiences are important to the effort to implement CAs in healthcare organizations and their application in care, as the adoption of new technologies often fails not because of the type of system, but because of the end user [13]. To realize the full potential of CAs and to ensure high-quality care in the future, it is therefore useful to elicit the opinions of physicians on the use of CAs in healthcare, as the systems are intended to support multiple areas of care. As a starting point for implementation, it is therefore necessary to understand physicians’ expectations and attitudes toward this technology [7].

Therefore, this study focuses on physicians’ perspectives on CAs. While the use of CAs in healthcare is widely discussed in the literature, their actual implementation remains rare. Recent studies highlight that most CA applications are still in pilot phases or limited to administrative and patient-facing tasks, with very few integrated into medical workflows [3, 4]. Given this early stage of real-world adoption, it is particularly important to understand how physicians assess the relevance, potential, and challenges of CAs for healthcare.

This exploratory study examines physicians’ expectations regarding the potential use of CAs in healthcare and identifies perceived barriers and facilitating factors relevant for future implementation. Given the limited real-world use of CAs, the study focuses primarily on expectations, attitudes, and anticipated implementation conditions. The discussion outlines implications that may inform future implementation initiatives.

## Methods

The study process and reporting is based on the Consolidated criteria for Reporting Qualitative research (COREQ) [14].

### Study design

In this qualitative study, physicians in Germany were asked about their expectations regarding the potential use of CAs in healthcare using semi-structured online interviews. The Framework Method [15, 16] was applied to examine physicians’ perspectives, expectations, and expected barriers and facilitating factors regarding the possible future implementation of CAs in healthcare. The Framework Method is a form of thematic analysis and was chosen because it provides a structure to consider data within and across interviews. It allows for an in-depth analysis of key topics across the entire data set, while preserving the individual context of the participants [15].

The study was reviewed and approved by the Ethics Committee of the University of Wuppertal (reference number: SK/AE 230314).

### Sampling strategy

In order to reflect different perspectives on the potential future implementation of CAs in healthcare, the principle of maximum variation sampling was applied in this study [17, 18]. The aim was to include as wide a range of relevant influencing factors and perspectives as possible in order to gain a comprehensive picture of the topic [19].

The sampling strategy was based on the theoretical replication logic according to Yin [20]. Cases were deliberately selected in such a way that both similar and different results could be expected based on theoretical assumptions. This made it possible to investigate whether central patterns and barriers in dealing with CAs from an expectations-based perspective emerge independently of individual or contextual differences, or whether specific differences between groups become apparent.

Two dimensions were taken into account for the selection: (1) individual characteristics such as age, gender, and previous experience with CAs, and (2) context-related variables such as medical specialty and level of care (outpatient/inpatient).

On the individual level, age and prior experience with CAs were assumed to shape the interpretation of and openness toward CAs, consistent with findings from innovation and technology acceptance theories [13, 21]. In contrast, gender was expected to have limited explanatory relevance for CA perceptions and thus served mainly to ensure heterogeneity rather than theoretical contrast. Physicians of different generations were interviewed, and two groups were formed for the current working population [22]: Age group 1 consisted of Generation X and baby boomers (born up to 1979), and Age group 2 consisted of Generations Y and Z (born from 1980 onwards). The characteristic “experience” was operationalized as whether or not the participant reported prior experience with a CA. Experience was assessed using a screening question that captured general CA use, irrespective of whether this experience occurred in clinical practice or in other contexts (e.g., private use, customer service, or non-medical domains). The broad operationalization of this criterion was intentional, as the study aimed to capture physicians’ perspectives on CAs regardless of prior clinical exposure, while acknowledging that practical clinical experience would likely yield more differentiated insights.

On the contextual level, we included physicians from different medical specialties and both inpatient and outpatient care settings. These dimensions were chosen because organizational context, workflow structure, and digital infrastructure are known to influence the perceived usefulness, feasibility, and potential integration of digital health technologies [20]. This sampling logic thus combined literal replication (expecting shared expectations across similar contexts) with theoretical replication (expecting variation between different generations, experience levels, and care settings).

The resulting sample aims for theoretical breadth, enabling a nuanced comparison of attitudes and expectations across contrasting professional and contextual conditions.

**Fig. 1:**
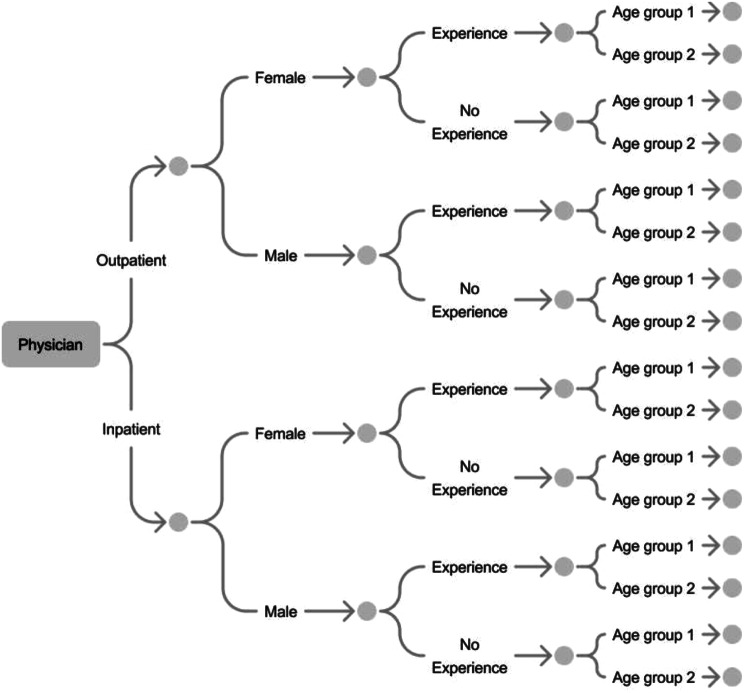
Sampling. Figure 1 shows the selection of participants based on the sampling criteria of physicians’ specialty, gender, experience and age

### Recruitment

Physicians practising in different medical fields and organizations (outpatient and inpatient care) were contacted specifically for this study. Postal recruitment focused on selected regions in North Rhine-Westphalia (urban and rural districts around Wuppertal) and Bavaria (greater Munich area), using publicly available contact information from the respective regional medical associations. As an additional recruitment strategy, a call for participation in the study was posted on the LinkedIn profile of the first author (MW) and was visible to physicians across Germany.

The physicians’ responses and informed consent were stored in a database from which we randomly selected those we wanted to interview, based on our sampling strategy. A total of 240 outpatient physicians and 30 key individuals, whom we used as gatekeepers to reach inpatient physicians, were contacted by post on August 1, 2023, to set up the database. We contacted 80 outpatient physicians from an urban district, 80 from a rural district and 80 from an urban district with a university. We also contacted key persons in the hospitals (mainly the medical directors of the hospitals) in the districts and asked them to pass on our request to their staff.

Interested physicians could register online to participate in the study until October 31, 2023. To do so, they had to complete a short questionnaire regarding their demographic information (Additional file 1) before they were assigned to one of our sampling categories. This questionnaire included a screening question on prior experience with CAs and an indication of the domain in which this experience was gained (e.g., healthcare or non-clinical contexts). In cases where this information was not specified, participants were contacted again and explicitly asked to clarify the context of their CA experience.

On November 1, 2023, interviewees were randomly selected from the database based on the information provided in the preliminary questionnaire and applying the sampling criteria mentioned above. The aim was to conduct one interview for each combination of sampling criteria, resulting in a minimum of 16 interviews. This sample size is suggested in the literature as an appropriate starting point for qualitative research [23]. We planned to continue recruitment after an initial analysis cycle if thematic saturation had not been reached [17, 24]. Despite extensive recruitment efforts and a high number of contacts, only one physician reported routine clinical CA use. As a result, the originally intended sampling strategy could not be fully implemented with regard to this dimension. Under these conditions, further targeted recruitment of clinically experienced physicians was considered unlikely to yield a sufficient number of participants. The final sample therefore represents a pragmatic adaptation of the original sampling strategy, reflecting current real-world conditions in which routine clinical CA use remains uncommon among physicians. Accordingly, the criterion “prior experience” was operationalized as general CA experience (including private and non-clinical contexts) rather than routine clinical implementation. Consequently, this study primarily reflects physicians’ expectations and hypothetical assessments rather than empirical implementation experiences. Thematic saturation was assessed with regard to physicians’ expectations, attitudes, and anticipated barriers and facilitators, rather than experiential knowledge derived from routine clinical implementation. Although the physician with clinical CA experience provided more differentiated and experience-based insights compared to participants without such experience, these insights remained singular and could not be systematically contrasted across a larger subgroup of clinically experienced physicians.

### Data collection

We followed a semi-structured interview guide to conduct the interviews. The interview guide (Additional file 2) was developed by MW and reviewed by JKN and SS based on the research question and previous work [2] applying the SPSS approach by Helfferich [25]. Questions with relevance to the research topic were collected, examined according to criteria for qualitative guides (e.g. openness of the question), sorted (e.g. content-related or chronologically) and subsumed into different question blocks [25]. The interview guide covered five main topics: (1) expectations of CAs, (2) preferred characteristics of CAs, (3) factors influencing the potential implementation of CAs, (4) further contextual factors to consider and (5) potentials and risks of CAs. In a recently published integrative review, we argued that, from the perspective of healthcare providers, it is important to distinguish between how a CA application is perceived when used by patients and how it is perceived by clinicians for their own purposes [2]. The five topics of the interview guide were therefore asked separately for the potential use of CAs by physicians and by patients. The interview guide was piloted during the first two interviews. Data of these interviews were included in the final sample, as no significant changes were required.

All interviews were conducted by MW (male, master’s degree in healthcare management, consultant) between November 2023 and January 2024. In regular meetings, MW and JKN (female, health economist, senior researcher, PhD, professor) discussed the progress of data collection and emerging challenges (i.e. difficulties in making appointments or encouragement of reflections). MW has already conducted qualitative interviews with healthcare professionals on the use of CAs in the healthcare sector and is experienced in qualitative data analysis [26]. MW and JKN have knowledge of the use of CAs in the healthcare sector based on previous research [2], and SS has knowledge in technology acceptance and implementation research on digital clinical decision support systems (CDSS) (female, public health scientist, post-doctoral researcher, PhD) [27, 28].

All interviews were conducted online via Zoom or Teams. The duration of the interviews was between 20 and 45 min. To achieve the aim of the study, a first run of 16 interviews (one per combination of sampling criteria) was conducted. After reaching inductive thematic saturation, an email was sent to all physicians in the database who were not selected to thank them for their willingness to participate and to inform them that the interview phase had been completed. Each participant was invited to contact MW after the interview by phone or email to share additional reflections. The characteristics of the participants were taken from the preliminary questionnaire that had to be completed at the time of enrolment.

All interviews were digital audio recorded. The audio recordings of the interviews were transcribed by MW using the simple transcription system of Dresing und Pehl [29]. To ensure the confidentiality of the data, the transcripts were cleaned of the names and identifiers of the participants.

### Data analysis

Data were analysed with the Framework Method, which involves an iterative analytical process consisting of seven stages: (1) transcription, (2) familiarization with the interview, (3) coding, (4) developing a working analytical framework, (5) applying the analytical framework (indexing), (6) charting data into the framework matrix and (7) interpreting the data [15, 16]. The Framework Method is a systematic and effective approach for analysing qualitative data in health research. It is a recommended method for analysing data from semi structured interviews with a limited number of topics and a sample size of approximately 10 to 20 participants [15]. It is important to keep in mind, however, that the analytical framework of this method has the specific purpose of managing and organizing the data and should be distinguished from the interpretation of the data and generation of themes [30]. To ensure the trustworthiness of our qualitative study, we applied established criteria including credibility, transferability, dependability, and confirmability [31]. Credibility was enhanced through independent coding and regular team discussions. Transferability was supported by providing rich descriptions of the study context and sampling strategy. Dependability and confirmability were ensured by maintaining an audit trail of methodological decisions and using the COREQ checklist to guide transparent and comprehensive reporting.

After conducting four interviews, MW and JKN initially analysed the four transcripts independently, line by line or section by section, and developed a set of codes. The coding was performed using an inductive approach. Both researchers then met to discuss, compare and adjust the codes to develop a working analytical framework, which seemed to provide a good fit with emerging issues in the data. The working analytical framework was then used by MW to index the subsequent transcripts immediately after the interviews with the help of the existing categories and codes and adjusted iteratively as interview data were indexed. In the analysis, interview data were systematically compared along the sampling dimensions to identify potential differences between subgroups. No particular computer-assisted qualitative data analysis software was used in coding the transcripts. In the following step, the data were summarized into matrices, further analysed to identify key elements, develop subthemes and identify connections between participants and categories. Matrix tables (spreadsheets) were organized in the software Excel. To ensure analytic transparency, we added a framework matrix example in the supplementary material (Additional File 3), which demonstrates how raw data were coded, categorized, and summarised into themes using the Framework Method. This interpretation step was mainly conducted by MW and supervised by JKN and SS. Researchers held regular meetings, incorporating elements of Bohm’s concept of dialogue [32], to discuss interpretations and move beyond descriptions of individual cases to develop themes that offered possible explanations for what was happening in the data.

In a subsequent analysis step, key facilitating and hindering factors for the potential future implementation of CAs in the healthcare sector were identified on the basis of the thematically condensed categories. Based on the coded interview data and the content summarized in the framework matrix, those statements were identified that explicitly or implicitly described conditions that promote or inhibit the expected use of CAs. The assignment was based on contextual relevance and interpretative weighting of the individual statements. The analytical framework of the Framework Method approach was retained and supplemented by a continuous comparison between cases and categories. The resulting systematization of the factors was brought together in a structured overview and served as the basis for the subsequent analysis of reciprocal relationships, dependencies and potential conflicts of interest between individual influencing factors.

Because the evaluation of an interview took place immediately afterwards, theoretical saturation could always be checked. After interviews 15 and 16, no new insights were identified, indicating that theoretical saturation had been reached and all relevant topics were covered. At this point, all remaining interested physicians were informed that no further interviews would be conducted.

## Results

A total of 53 physicians registered in our database to participate in the study. Sixteen participants were selected at random in a first run using our sampling method. We interviewed eight physicians who deliver outpatient care in a private practice and eight physicians who work in hospitals and deliver inpatient care. The participants were on average 44.2 years old. The youngest physician was 28 years old, and the oldest interviewee was 63 years old. Although half of the participants had some prior experience with CAs, these experiences mainly stemmed from private contexts (e.g., e-commerce or customer support). Only one physician had used a medical CA (Ada Health) in medical practice. Table 1 presents the self-reported demographic information.

**Table 1:** Participants’ sociodemographic characteristics (*n* = 16)

Characteristic	*n* (%)
**Sex**	
Female	8 (50)
Male	8 (50)
**Age (years)**	
28–44	8 (50)
45–63	8 (50)
**Health professional role**	
** Physician working in inpatient care**	8 (50)
Internal medicine	3 (37.5)
Neurology	1 (12.5)
Rheumatology	1 (12.5)
Surgery	1 (12.5)
Urology	1 (12.5)
Oncology	1 (12.5)
** Physician working in outpatient care**	8 (50)
General practitioner	4 (50)
Gynaecology	1 (12.5)
Neurology	1 (12.5)
Rheumatology	2 (25)
**Experience with CA**	
** Yes**	8 (50)
Experience in the medical field	1 (6.25 of total; 12.5 of experienced)
Experience in the private field	7 (43.75 of total; 87.5 of experienced)
** No**	8 (50)
**Technological affinity**	
Technologically savvy	10 (62,5)
Not technologically savvy	6 (3.5)

Baseline characteristics. *n* denotes the number of participants, % proportions

Five main themes were identified that correspond to the purpose of the study. Based on the five topics, we then describe the key facilitating and hindering factors that, according to the interviewed physicians, may influence a potential future implementation of CAs in the healthcare sector. As the interviews were conducted in German, the original quotations are also in German. For the purpose of supporting the results, they were translated into English by MW. The translation was verified by JKN. Additional file 4 shows the original quotations in German and the corresponding English translation.

### Theme 1: Regulatory, structural and risk-related barriers to the implementation of CAs

Our analysis of the interviewed German physicians shows that, within the German healthcare context, current regulations and conditions can hinder progress in the healthcare sector and that the full potential of available technologies for patient care is not being realized. According to the physicians interviewed, it is therefore urgently necessary to change these regulations and conditions.

*Healthcare is a budget-driven system*,* and we clearly lack the budget for innovations. But of course*,* it also has to do with people. And with conditions such as regulations*,* data protection and fears that you might do something wrong. […] There are also various other reasons*,* above all the fact that we are chronically understaffed and have no time to try out and implement new technologies.* (IP-8)

Most respondents explained their lack of real-world application by referring to data protection regulations, insufficient digital infrastructure, and limited financial resources. In this context, it is notable that participants in Age group 1 primarily mentioned data protection, and the younger participants most frequently mentioned the lack of modernity of hospitals as an obstacle to the use of CAs.

*I think it’s just too future-oriented. Most hospitals are not that modern yet. If they’re not even digital*,* then I don’t think you can integrate CAs and AI yet.* (IP-9)

Participants further highlighted technical incompatibilities and lack of interoperability as central challenges. Since each hospital or practice uses different software systems, integrating a CA into existing structures was expected as extremely complex.

*The integration into existing systems is the biggest challenge. The CA must be adaptable to different systems and easy to integrate into existing systems.* (IP-10)

Alongside these structural problems the participants identified a number of risks associated with the use of CAs in the healthcare system (summarized in Additional File 5). They expressed concerns regarding liability, data protection, and potential misuse of data. They criticized that, despite ongoing discussions about digitalization, legislation and policy-making remain hesitant to create enabling conditions for technological innovation. For a potential future establishment of CAs in the healthcare system, the physicians considered it necessary to address the risks mentioned, as these were perceived as decisive inhibiting factors. In particular, they hoped that the legislator would find timely solutions to the legal problems (liability and responsibility for errors) and data protection.

*I think it is now up to legislators to develop solutions and have the courage to do something.* (IP-6)

Several physicians pointed out that the debate about risks such as data protection often serves as a convenient excuse to block innovation rather than representing a true barrier.

*The issue of data protection is often put forward as a reason for new innovations*,* but it can honestly all be solved.* (IP-3)

They also hoped for a risk–benefit analysis from the legislator that focuses on the benefits (people’s health and lives) rather than the risks of technologies such as CAs. One physician pointed out that not using data and technology also costs lives, possibly more than the existing risks of systems such as CAs.

Overall, physicians expected the rigid regulatory framework, unclear legal responsibilities, and lack of technical interoperability as the main barriers to the sustainable establishment of CAs in healthcare. This shows that, according to the physicians, a number of obstacles still need to be removed in order to establish CAs sustainably. Despite these regulatory, structural, and risk-related barriers, physicians also expressed a strong awareness of the pressing challenges within the healthcare system and the potential value of technological innovation. While concerns about data protection, interoperability, and legal responsibility dominated their critique, many interviewees simultaneously recognized that CAs could play an important role in addressing current strains on healthcare delivery. The following theme therefore describes how physicians envision the potential benefits and concrete use cases of CAs in clinical practice.

### Theme 2: Expected structural and system-level potentials and use cases of CAs

Physicians in our study highlighted a wide range of anticipated structural and system-level benefits and concrete use cases through which CAs could support clinical practice, improve efficiency, and would make work easier for medical staff and relieve their workload. They agreed that relief was urgently needed.

Despite the existing challenges, all participants expressed a fundamentally positive attitude toward CAs and highlighted their potential to improve medical care. Physicians agreed that CAs could help manage increasing workloads, compensate for staff shortages, and maintain care quality.

*CAs will play an increasingly important role in healthcare in the future and will be an integral part of healthcare provision*,* because we physicians will have to care for more and more patients*,* and then we will need the support of such chatbots.* (IP-5)

A recurring topic was the expected efficiency gains through automation of administrative and routine tasks. Nearly all physicians identified documentation as the most time-consuming and burdensome aspect of their work and hoped that CAs could significantly reduce this effort.

*I’m hoping for the greatest support with documentation. I have the feeling that I could treat three times as many patients if I didn’t have to document as much as I do.* (IP-16)

Participants envisioned that CAs could take notes during consultations, write physician letters, prepare invoices, or generate summaries for medical records. The physicians expected to save time by using CAs for support. They wanted to use the time gained for more intensive and individualized patient treatment. In addition to administrative tasks, physicians saw diagnostic support and symptom assessment as promising use cases, especially in initial triage or pre-consultation phases. They anticipated that CAs could reduce unnecessary visits, save costs, and streamline patient flows.

They also noted that the use of CAs by patients would lead to a pre-selection of patients and thus reduce the burden on the healthcare system.

*[…] the hope is also faster access to care and the appropriate access. In other words*,* the patient is told directly that these are the right specialists for their complaints*,* and not everyone is sent to the emergency room*,* but sent directly to the right care area […]. (IP-9)*

Furthermore, the physicians assumed that the use of technologies such as CAs could compensate for the prevailing and impending shortage of skilled workers. The participants thought that technology would not be a substitute for these jobs but could fill gaps where there is a shortage of skills. In one physician’s opinion, “[…] *routine and simple tasks will be performed by CAs in order to counteract the shortage of specialists.*” (IP-15) Participants agreed that CAs in healthcare currently fulfil a supportive role.

Importantly, our results indicate that awareness of the capabilities and implementation possibilities of CAs emerges only through practical use in medical practice. The only participant who had already tried a medical CA (Ada Health) in daily practice saw the potential and applications of CAs that the other participants did not think of due to the lack of practical benefits. This participant was also able to provide quantitative figures on efficiency gains from practical experience. This finding underlines the importance of the trialability of CAs under real conditions and in daily practice.

While physicians primarily emphasized the efficiency and workload-reducing potential of CAs, their reflections also extended to how these technologies might influence the quality and safety of medical care. While the aspects described here relate to structural and system-level implications of CAs, physicians also reflected on how CAs might affect medical treatment itself. The next theme therefore examines physicians’ expectations on treatment-related benefits and risks.

### Theme 3: Quality increase and risks in treatment through CAs

The physicians interviewed agreed that the introduction of CAs could potentially lead to improvements in the quality of daily care, according to the physicians’ expectations.

*CAs can significantly increase the efficiency of treatment and diagnostics*,* as the CA can also take into account non-obvious and rare diseases*,* summarize the patient’s medical history*,* laboratory values and past history in a short time*,* identify drug intolerances and call up comparative cases with a similar course.* (IP-2)

The physicians also expected that the support would lead to faster diagnosis. They pointed out that, as a personal assistant, a CA can also convey a sense of security, which is particularly important for young physicians with little treatment experience. All physicians interviewed assumed that the use of CAs would increase the quality of care, as the support of a CA in daily care could guarantee the best possible individualized therapy for patients.

However, the physicians warned that if technology replaced physicians or if CAs were misused by physicians or patients, there would be a loss of quality of care.

*As long as CAs are an extra system*,* I don’t think we will have a loss of quality. But as soon as they replace the medical staff*,* it will automatically lead to a loss of quality.* (IP-16)

The participants saw the danger that, in the future, medical staff might rely solely on the solutions proposed by CAs and follow them blindly, resulting in the profession forgetting how to retrieve knowledge in acute situations and the loss of knowledge in the long term. Conversely, a CA that always produces different diagnoses or treatment suggestions could lead to medical staff losing confidence in their own abilities. Another concern raised was that CAs could lead to overdiagnosis and therefore more time-consuming treatments, as the technology may point to multiple suspected diagnoses or may not be able to draw on experience from previous treatment decisions that take into account patient preferences. If all suspicious diagnoses, however minimal, are followed up, the use of CAs would lead to increased costs. The physicians also assumed that patients would be overdiagnosed by the use of CAs. They feared that some patients would have every minor ailment checked, which could lead to hypochondria. Some physicians therefore feared that the use of CAs could create more sick people, which would place an immense financial burden on the healthcare system.

Above all, the physicians feared that the use of CAs as symptom checkers would diminish their medical opinion and authority. They expected that some patients would present with a diagnosis of CA and want to be treated according to the technology’s suggestion, even if the diagnosis was wrong or there was a more appropriate therapy. For the physicians interviewed, it is therefore important that patients are clearly informed that a CA is only a supportive tool and that the physician remains the final decision-maker in medical matters. Furthermore, some physicians feared that some patients would no longer visit medical facilities, because they would only consult a CA about their medical concerns and only trust technology.

The results show that physicians expect that CAs could contribute to diagnostic support and treatment quality, but they emphasized that their safe and meaningful use would require clear medical accountability and robust safeguards to avoid clinical risks and ensure patient safety. Beyond questions of clinical quality and safety, several participants linked the introduction of CAs to broader transformations of the medical profession itself, which is described in the following theme.

### Theme 4: Changes in the medical profession due to CAs

The physicians believed that the healthcare professions, and the medical profession in particular, would change through the use of technologies such as CAs.

*The entire healthcare system and our day-to-day work will change through the use of technology. In order for us to be able to handle the systems and understand how they work*,* we also need more IT-oriented training for medical professions.* (IP-5)

The physicians also anticipated a change in patient care through CAs.

*We will be treating more and more of our patients at home […]. This can also be done using technology such as CAs*,* which collects the information and forwards it to me in a structured way.* (IP-5)

They explained that the use of CAs appears to be particularly valuable for elderly patients who cannot make it to the physician’s office regularly and for people who live in rural or underserved areas. With technology, such people can be closely monitored at home rather than being admitted to hospital.

Participants also pointed out that medical knowledge is now so vast and growing that it is impossible for a physician to know and consider everything. With a CA as a support tool, all medical knowledge would be available, and the physician would always be up to date, which would significantly improve the quality of care. Because CAs are fact-based and can bring together large bodies of knowledge, the physicians believed that they could outperform humans in some areas, such as diagnosing multiple and potentially interrelated aspects of a therapy or interpreting laboratory values.

Building on these anticipated transformations in professional practice, physicians also outlined what characteristics and technical requirements CAs must fulfil to be accepted and effectively integrated into care. The following theme summarizes the key expectations and design features that participants considered essential for trustworthy, user-friendly, and sustainable CA implementation.

### Theme 5: Physicians’ expectations of CAs

**Fig. 2:**
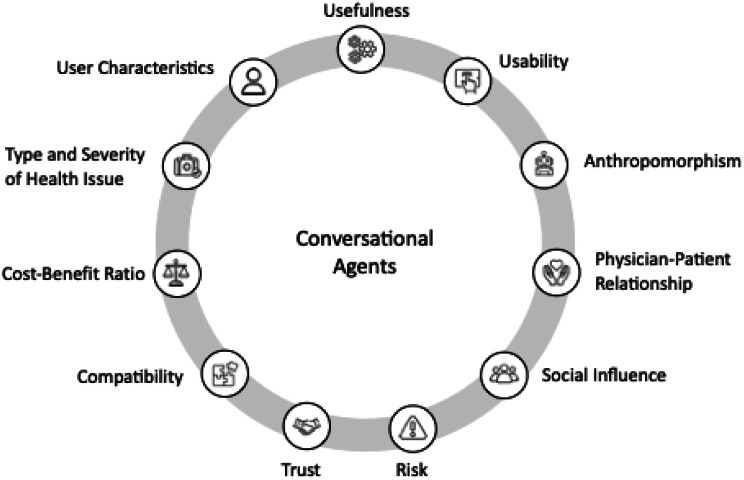
Expected CA implementation requirements. The figure illustrates the 11 characteristics that physicians described as important for the acceptance and use of CAs in healthcare. Note: User characteristics and type/severity of health issue are contextual factors influencing acceptance and use, not intrinsic CA characteristics

A total of 9 characteristics were identified that CAs in the healthcare sector should possess, according to the interviewed physicians, in order for the systems to be accepted and used in the long term. In addition, physicians emphasized that user-related factors (such as age, affinity for technology, previous experience with CAs) and contextual factors (such as type and severity of the health issue) could play an important role in the acceptance and use of CAs, but are not characteristics of the CA itself. Figure 2 shows a summary of all the mentioned characteristics.

All participating physicians emphasized that the design, transparency, and reliability of CAs are decisive for their acceptance by both physicians and patients. Building trust in the system emerged as one of the most critical preconditions for potential future implementation.

The two characteristics most frequently mentioned by physicians were performance reliability and usability. Participants underlined that CAs must operate consistently and without technical errors in order to be accepted in clinical practice.

*The CA must be a permanently functioning system that has practically no technical failures and always fulfils its purpose. If it doesn’t*,* I wouldn’t use it and wouldn’t recommend it to my patients*. (IP-5)

In addition, the source from which the CA draws its information and how it presents it was decisive for all physicians. In this context, several physicians explicitly expressed concerns about the trustworthiness of CAs and the origin of the information provided. They emphasized that AI-based systems in particular can potentially provide incorrect or misleading answers, which could have serious consequences for patient safety in healthcare. The transparency of data sources, the traceability of decision-making logic, and regular content reviews were cited as essential prerequisites for the safe use of CAs. Several physicians emphasized that the risks of incorrect answers and a lack of transparency in CAs can only be managed through clear quality standards, regular content reviews, and human oversight.

The system should also be *“user-friendly and operable by everyone”* (IP-8) so that users like to use it and perceive it as a real alternative. In addition, the CA should be individually customizable by the user in all functions and appearance, as *“everyone has different tastes*,* preferences and needs” (IP-12).*

The relationship between the user and the CA and the factors influencing trust and risk in the relationship were also decisive factors for all the physicians interviewed and should be taken into account when introducing CAs in the healthcare system. According to the physicians, human qualities such as empathy and trust in the system are important to achieving such a bond between user and CA. The physicians wanted a relationship with the CA that was comparable to that of a work colleague. For them, trust and reliability were decisive characteristics. The CA should therefore *“be reliable*,* i.e. make correct diagnoses*,* suggest the best possible treatment and have information from a credible and evidence-based source. At the same time*,* I don’t always want to have to question everything; I want to have a sense of security”* (IP-6).

Many of the physicians interviewed also pointed out that cost-effectiveness has an influence on the adoption of CAs. For them, it was important that the systems offered added value, and they weighed up the perceived benefits of the system against the costs incurred.

*A classic cost–benefit analysis is also carried out here. However*,* the costs of introduction and benefits must not be too high*,* otherwise it will of course not be introduced due to our limited budget.* (IP-4)

The physicians interviewed assumed that user-related factors such as age, affinity for technology, previous experience with CAs and reservations were decisive for the acceptance and use of CAs.

This finding shows that for broad acceptance and use of CAs in the healthcare sector, the systems must have a variety of characteristics. It is also important to consider which user group the CA is aimed at, as each of them has different requirements for the system.

### Facilitating factors versus hindering factors for potential future implementation

Key enabling and hindering factors for the potential future implementation of CAs in the healthcare sector were derived based on the analysis of the five identified categories and the systematic evaluation of the interviews conducted as part of this study. The following table summarizes these expected factors systematically and serves as a basis for analysing their mutual relationships and potential conflicts of interest.

**Table 2:** Expected facilitating and hindering factors for a potential future implementation

Expected facilitating factors	Expected hindering factors
• Positive attitude of physicians • Potential to reduce workload for physicians • Time savings and efficiency gains • Improved opportunity to access the healthcare system • Experienced/anticipated ability to improve patient empowerment and self-determination • Quality improvement • Individual adaptability and user-friendliness • Trust and empathy through human-like interaction • Potential for cost reduction • High acceptance among physicians • Broad range of possible applications • Knowledge expansion and decision support • CAs as a solution to rising patient numbers and staff shortages • Future viability and necessity	• Regulatory and legal requirements • Technological barriers and lack of interoperability • Structural barriers and lack of digital infrastructure • Lack of practical experience • Risk of overdiagnosis • Felt loss of medical authority • Resistance due to age and technology affinity • Lack of empathy and social connection • High implementation costs and unclear return on investment • Lack of trust in data sources and transparency of results • Lack of time for implementation and experience • Insufficient budget • Quality loss due to misuse

Table 2 provides an overview of the key facilitating and hindering factors that physicians perceive as relevant for a potential future implementation of CAs in healthcare

According to the physicians interviewed, any potential future integration of CAs in the healthcare sector would depend on a complex interplay of technical, organizational, structural, and social factors. Conducive expectations such as increased efficiency, relief for physicians, patient empowerment, and improved access to care were contrasted with a wide range of anticipated barriers whose interactions were perceived as shaping the feasibility of future implementation efforts.

The analysis indicated that physicians’ general attitudes toward digital innovations were described as an important aspect shaping their expectations about CAs. Many interviewees expressed openness toward such technologies, often in light of increasing workloads, rising patient numbers and shortages of medical staff. From the physicians’ perspective, opportunities to try out CAs in real world clinical practice, referred to as trialability, were considered essential for moving beyond hypothetical assumptions and for developing more concrete, experience-based expectations regarding both potential benefits and risks. Participants emphasized that many effects of CA use that are discussed in the literature, whether related to efficiency, workflow integration, or clinical decision support, can only be meaningfully assessed through hands on use under routine conditions. In the absence of such opportunities, expectations remained largely speculative. Physicians perceived trialability to be currently constrained by legal uncertainties, the lack of pilot projects, and structural barriers such as outdated IT infrastructures. Accordingly, trialability emerged as a key factor shaping physicians’ assessments of the usefulness and feasibility of CAs, while its limited availability was seen as a central obstacle to informed evaluation and acceptance.

From the physicians’ perspective, CAs could also offer potential benefits on the patient side, such as providing low-threshold access to health information, supporting symptom assessments, and potentially strengthening health literacy. According to the interviewees, such functions might help reduce pressure on healthcare services while responding to patients’ increasing need for personalized, around-the-clock support. At the same time, physicians also anticipated possible risks, including overdiagnosis, misuse, or reduced engagement with physical care if patients misunderstand the role of CAs or overestimate their capabilities. This could create a tension between empowerment and responsibility, which would need to be addressed through clear communication frameworks and quality-assured guidance for users.

In the physicians’ view, building trust is crucial for acceptance, among both physicians and patients. Factors such as user-friendliness, transparency of data sources, evidence-based decision-making logic and anthropomorphic design (e.g. through avatars or empathic communication styles) are conducive to trust development. Nevertheless, the physicians anticipated that technology-related reservations, particularly among older or less technically oriented user groups, may persist even when usability is high. They also expressed concerns that limited transparency in algorithmic processes might make it more difficult for users to develop trust in such systems.

The physicians further emphasized that various structural, technological and regulatory barriers are anticipated. They mentioned data protection concerns, unclear liability issues, the absence of clear legal standards and the often outdated and heterogeneous IT infrastructure as factors that, in their view, could complicate the future integration of CAs into existing healthcare systems. Economic considerations such as high initial investments and uncertain profitability assessments were also described as potential obstacles, particularly within heavily budgeted healthcare systems. In addition, many participants felt that time and personnel resources for testing new technologies in clinical practice are limited, which they perceived as particularly challenging given the urgent need for workload relief.

In the interviews, the physicians described the potential future integration of CAs in healthcare as a complex issue that goes beyond purely technological considerations. The thematic analysis showed that the participants consistently associated this topic with structural, legal, organizational, and user-related conditions. The physicians repeatedly emphasized that, in their view, it was important to address these conditions, for example by removing structural barriers, clarifying legal and organizational frameworks, taking into account the needs of both physicians and patients, and promoting trust-building and user competence. Taken together, these themes suggest that the integration of CAs is a complex process characterized by interrelated factors that are expected to influence the feasibility of future implementation efforts in routine care.

## Discussion

The aim of this study was to understand how physicians expect the potential use of CAs in healthcare and to identify barriers and facilitators that may need to be addressed in future implementation efforts. The analysis revealed five key themes related to how physicians expected the potential use of CAs in healthcare.

Two main subgroup differences were observed. The most notable distinction concerned prior experience with medical CAs: the only physician with real-world CA use provided more differentiated, experience-based insights, identified additional use cases, and even quantified potential efficiency gains, whereas physicians without such experience mainly discussed hypothetical potentials and barriers. This highlights how real-world exposure shapes expectations regarding usefulness and limitations and underscores the importance of trialability in technology adoption and innovation diffusion [20, 21, 33]. A second difference emerged between age groups. Younger physicians more often emphasized structural and technical barriers such as outdated IT infrastructure, while older physicians focused on data protection and liability concerns. These patterns likely reflect generational differences in digital familiarity and professional socialization [21, 22]. However, beyond these nuances, no appreciable differences were identified in the expectation towards CAs across the other sample characteristics, including gender, and physicians’ specialty (outpatient or inpatient). This result suggests that the central challenges and potentials of CAs in physicians anticipated future practice are largely expected independently of most individual or contextual characteristics. The finding that key themes emerged consistently across these diverse groups indicates a high degree of pattern stability and supports the robustness of the reported expectations. At the same time, consciously including different sampling dimensions proved methodologically useful, as it strengthened the credibility and transferability of the findings and demonstrated that the observed patterns are relevant to a broad group of physicians. Nevertheless, this result underscores the need for future studies with even greater heterogeneity or a targeted focus on specific contexts to further explore potentially finer-grained variations.

### Discussion of the results and comparison with existing literature

The results of this study show that physicians expect CAs to be promising tools that may help to reduce workload, increase efficiency, and improve access to care—especially in light of staff shortages and rising patient volumes. These findings are consistent with existing research highlighting the potential of CAs to support clinical processes and promote patient-centred care [3, 5].

A key finding of this study is the limited practical experience with CAs among the physicians surveyed. Despite extensive recruitment efforts and a large number of potential participants, only one person could be identified who had actually used a CA in everyday clinical practice. This finding is consistent with recent studies showing that CAs have so far only been used sporadically in healthcare and that most applications are still in the pilot stage or in administrative or patient-related areas [3, 4]. The low practical use of CAs thus not only reflects the current care situation, but is itself an important finding of this study. The expectations, attitudes, and mentioned potentials and barriers of those physicians who have not yet gained any experience with CAs are nevertheless highly relevant, as they represent the main target group for future implementation efforts. Our results therefore emphasize that implementation strategies should not only address technical and organizational barriers, but also specifically consider the information needs, concerns, and expectations of those physicians who have not yet had any contact with CAs. Future studies should specifically include physicians with practical CA experience as soon as these technologies are more widely used in order to further optimize implementation processes and evaluate their real-world benefits in routine practice.

Based on the thematic analysis, key facilitating and hindering factors for the potential implementation of CAs were identified. Participants showed a generally positive attitude toward technology-assisted medicine, reflected in their openness to CAs and their expectation that these could reduce administrative workload, support chronic care, and improve access for patients - particularly for simple medical concerns. A central expectation was the potential diagnostic use of CAs, especially for differential diagnosis. Existing studies indicate that CAs can achieve diagnostic accuracies comparable to human physicians and may effectively support clinical decision-making [34–37], with one study reporting that over 90% of correct diagnoses appeared within CA-generated differential lists [36]. Physicians in our study attributed such potential to CAs and expected them as helpful assistants for structuring information and supporting diagnostic reasoning.

A primary concern of the interviewed physicians was the expectation of obtaining relief from administrative tasks, particularly documentation, which they reported often having to complete outside regular working hours due to high workload. Previous studies indicate that administrative activities account for around 25% of physicians’ working time, corresponding to approximately two hours per day [38, 39], underlining the relevance of this burden. Several studies suggest that CAs have the potential to reduce documentation time and associated costs. For example, CAs were shown to reduce documentation time in urology by 44% while achieving an accuracy of over 95% in generating medical texts and discharge letters [40]. Other studies also report time savings through CA-supported anamnesis and improved consultation efficiency [3, 41]. Consistent with these reported potentials, physicians in our study most frequently mentioned documentation, symptom assessment, and patient empowerment as key anticipated areas of application for CAs and associated workload relief, which they expected would allow for more personalised patient care.

Despite these opportunities, substantial hurdles to potential implementation persist. These include legal uncertainties such as data protection and liability issues, technical challenges such as a lack of interoperability and inadequate digital infrastructure, and structural barriers such as a lack of time and resources. Some physicians believe that data protection concerns are sometimes used as an excuse to prevent digital innovation, which is also discussed in the scientific literature [3, 42]. Beyond these practical challenges, the findings highlight ethical concerns that extend beyond managing technical risks. Physicians emphasized that AI systems should not assume decision-making authority, as this could undermine clinical responsibility and accountability. Several interviewees also expressed concern about potential deskilling, fearing that overreliance on CAs could weaken clinical reasoning and long-term professional competence. These expectations underscore the need for implementation strategies that ensure meaningful human oversight and prevent cognitive dependency through clear governance mechanisms and transparent system behaviour [8, 43].

In addition, the lack of healthcare providers and limited accessibility, particularly in rural and underserved areas, represent ongoing challenges for healthcare systems [5]. Studies have shown that people in rural areas have to travel significantly further to see a physician than city residents. Long distances are particularly difficult for older people and people with serious injuries [44]. The physicians interviewed also mentioned that more and more people will have to be treated at home in the future, because they live in an underserved area or cannot make it to a practice regularly due to their age, for example. Physicians in our study believed that CAs could potentially contribute to improving access to care, particularly in underserved areas. This expectation is consistent with prior studies highlighting the potential of CAs to increase health information accessibility [45, 46].

A comparison with established implementation and innovation frameworks such as the updated CFIR [47] and the NASSS-CAT framework [48], it becomes apparent that many of the factors identified in this study such as compatibility with existing systems, user-friendliness, cost-benefit ratio, regulatory and structural barriers represent generic challenges for the introduction of innovations in healthcare. Aspects such as acceptance, training needs, and adaptability are also relevant in many innovation contexts and are described as key determinants in the frameworks mentioned above. For technology-based innovations, especially digital solutions, factors such as data protection, interoperability, technical reliability, and integration into existing digital infrastructures are also becoming increasingly important. These aspects are also explicitly addressed in the NASSS-CAT framework and are also reflected in the statements of the physicians surveyed [47, 48]. However, anthropomorphism, working alliance, empathy, and trust are particularly relevant topics for CAs in healthcare. Our results show that physicians attach great importance to the quality of the relationship between the user and the CA, an aspect that has received little attention in traditional innovation frameworks to date. The ability to establish a trusting working relationship between the CA and the user through anthropomorphic design elements (e.g., avatars, human language, empathetic communication) is considered crucial for the acceptance and sustainable use of CAs [49]. This clearly distinguishes CAs from other digital innovations, where interaction is usually less dialogical and relationship-oriented. In order to promote emotional closeness and trust, we recommend considering providing users with a health CA with an avatar, with which the patient can establish a connection.

The systematic consideration of the factors and their interdependencies indicates that the potential implementation of CAs is not a purely technological introduction, but a complex adoption process that operates on several levels. In the following section, the present findings are integrated within an overarching theoretical model, thereby facilitating a more profound comprehension of the empirical observations within the context of this framework.

### Theoretical integration of the results

The empirical results are integrated into an established theoretical model for innovation research in order to derive starting points for the potential future implementation of CAs in the healthcare sector. Specifically, as a comparison we use Rogers’ Diffusion of Innovation Theory, which is one of the best-known social science theories for the implementation and adoption of new innovations. This theory attempts to explain how new ideas or innovations (such as CAs) are adopted. It assumes that five characteristics of an innovation influence adoption: (1) relative advantage, (2) complexity, (3) compatibility, (4) trialability and (5) observability [33].

According to Rogers’ Diffusion of Innovation Theory, innovations that have a clear added value in terms of effectiveness or cost-effectiveness are more easily adopted and sustained (relative advantage). Our results show that many of the factors identified as beneficial by the physicians interviewed are consistent with this concept. Expected benefits such as reduced workload, increased efficiency, better access to care and time savings - both in the administrative area and in patient communication or decision support in the diagnostic and therapeutic process - were mentioned particularly frequently. In addition, cost aspects were discussed intensively by the interviewed physicians at several points during the study. According to the physicians, CAs are expected as having the potential to achieve positive economic effects in the long term within the German healthcare system. From their perspective, this potential is primarily linked to the automation of administrative processes and the optimisation of clinical workflows, which they expect could lead to a more efficient use of resources. At the same time, the physicians emphasised that current budgetary framework conditions in the German healthcare system pose considerable challenges, as they allow only limited financial leeway for investments in innovative technologies. In their view, this discrepancy between the anticipated economic and organisational benefits of CAs (e.g. cost reduction, process optimisation, and potential improvements in care processes) and the restrictive financial resources represents a significant barrier to implementation [3, 40].

Furthermore, it is important that the relative advantage is visible to the intended users (observability) [33]. The physicians interviewed reported that performance expectancy in the form of usefulness and effectiveness was an indispensable requirement for the adoption of CAs, and they expected the benefits and potential of using CAs in healthcare for themselves and their patients. Previous studies have shown that both physicians and patients recognize the effectiveness and benefits of CAs in healthcare [11, 50].

However, the relative advantage alone is no guarantee of the adoption of an innovation [51, 52]. If the results of an innovation are highly uncertain and users perceive it as risky, the innovation is less likely to be adopted [42]. Our results show that even with a positive attitude towards CAs, legal uncertainties, technical barriers and a lack of resources can significantly hinder the potential implementation process. The main risks of CAs reported by the physicians interviewed and the literature are legal problems (liability and responsibility in case of errors) and jeopardized patient safety due to incorrect diagnoses [2, 3]. These risks should be addressed through a combination of legislative action, responsible system design by developers, and transparent dialogue with the medical community to ensure safe, meaningful, and ethically sound use of CAs. In addition, the concerns and hesitancy of physicians as key stakeholders should be taken into account when prioritizing the relative advantages of CAs. Beyond these expected regulatory challenges, our findings also suggest an underlying tension that was only implicitly expressed by participants: while physicians frequently described regulations and legal requirements as obstacles for the introduction of CAs, these same frameworks also serve as essential safeguards for professional accountability, patient safety, and organizational responsibility [3, 8, 12]. Several interviewees referred to the high perceived liability and responsibility associated with clinical decisions, indicating that clear legal standards are not only restrictive but also protective in ensuring that physicians remain on safe professional and legal grounds [8]. This ambivalence highlights an important trade-off: the desire for more flexible conditions that would enable technological innovation, and the simultaneous reliance on regulatory structures that guarantee secure and ethically responsible practice [3, 12]. Understanding how physicians navigate this tension is crucial for future implementation efforts, as it suggests that regulatory reform should not merely remove barriers, but carefully balance innovation-enabling flexibility with indispensable protective functions.

In addition to the relative advantage, innovations that are perceived as easy to use are more easily accepted and adopted [33]. Numerous studies have identified perceived usefulness and intuitive usability as key predictors for the actual use of new technologies [53, 54]. Our study also indicates that a user-friendly design, intuitive interaction and low access barriers are essential prerequisites for the widespread use of digital solutions. In the healthcare sector in particular, performance expectations and user-friendliness were identified as the two key factors influencing the acceptance of CAs [2].

Perceived complexity can be reduced through practical experience and demonstration. At the same time, the relative advantage of practical use can be perceived by users [55]. Since innovations require time, energy and resources, innovations that can be tried out before they are fully introduced are more likely to be adopted [33]. For the sustainable establishment of CAs in healthcare, therefore, it is important that the systems can be tested by physicians and patients in a timely manner. The opportunity to try out a technology in everyday life can reduce uncertainty, strengthen acceptance and make positive effects tangible. It has been found, however, that CAs have almost exclusively been tested in controlled environments that do not simulate realistic interactions in clinical practice [56, 57].

Compatibility with existing systems, the fifth characteristic of Rogers’ Diffusion of Innovation theory, was particularly important for the physicians interviewed. According to them, it represents one of the greatest challenges for the potential future establishment of CAs in healthcare. Evidence shows that the more compatible the innovation, the greater the probability of adoption [33, 51]. The participants emphasized that meaningful use of CAs is only possible if the systems can be seamlessly integrated into existing IT infrastructures. Currently, a lack of interfaces, fragmented system landscapes and unclear responsibilities are perceived to prevent effective integration. Studies also show that a lack of compatibility is a common reason for the failure of technical innovations in the healthcare sector [58].

Overall, our findings suggest that physicians perceive the potential implementation of CAs in healthcare as a complex and multidimensional adoption process. While Rogers’ diffusion theory acknowledges the relevance of social systems and contextual influences, the physicians in our study described the healthcare environment—with its regulatory constraints, safety requirements, professional autonomy concerns, and fragmented IT infrastructures—as creating particularly intricate interdependencies between the five innovation characteristics. This complexity became evident in their simultaneous recognition of potential benefits, such as workload reduction, alongside persistent barriers, including limited interoperability and unclear legal responsibilities. These perceptions illustrate how the characteristics of innovations interact in practice within healthcare settings.

Across the interviews, physicians emphasized several conditions they considered important for enabling future implementation efforts. These included opportunities for practical testing (e.g. pilot projects), clearer regulatory guidance, improved digital infrastructure and interoperability, targeted training, and adequate resource provision. From their perspective, addressing these aspects would be necessary for any sustainable integration of CAs in routine care. They also highlighted the importance of involving policymakers, developers, healthcare providers, and patients to ensure that diverse needs and perspectives are reflected in future implementation strategies.

### Limitations

This study has several limitations that must be considered when interpreting the findings. The sampling approach may have introduced a self-selection bias. Physicians who chose to participate may have had a stronger pre-existing interest in digital innovations or CAs, which could have led to a more favourable or more critical view of their expected use than is representative of the general physician population. Furthermore, only physicians working in Germany were interviewed about their expectations of CAs in the healthcare system. Thus, the responses were expectations shaped by the specific regulatory and organizational context of the German healthcare system. It is therefore questionable to what extent our results are transferable to healthcare systems in other countries. Future cross-national comparative studies would help distinguish between universal CA implementation challenges and those specific to particular healthcare contexts. Moreover, all interviews were conducted in German and subsequently translated for analysis and reporting. While great care was taken to preserve the meaning of the statements, nuance and emphasis may have been altered in the translation process. In addition, all interviews were conducted remotely (via Zoom or Teams), which could have affected the interaction. On the one hand, it may be harder to connect with interviewees remotely, but on the other hand, people may feel more anonymous and comfortable with sharing information.

Furthermore, a key limitation concerns the level of clinical experience with CAs among the participants. The screening questionnaire recorded whether participants already had experience with CAs and in which area of application this experience had been gained. However, the recruitment process showed that this experience was predominantly related to private or experimental use and not to professional application in routine everyday medical practice. Only one participating physician reported practical experience with a CA in daily medical care. As a result, the assessments of most participants were based more on expectations than on their own experience with the application. This limitation is partly due to the broad operationalization of prior experience with CAs in the screening process and partly reflects the early stage of practical implementation of CAs in clinical care.

## Conclusion

The findings suggest that, according to physicians, future implementation efforts would likely need to consider multiple dimensions that equally considers technical performance, regulatory frameworks, integration into clinical workflows and human factors such as acceptance, trust and user competence. It is essential that CAs support decision-making without undermining clinical judgment or professional development. Trust in such systems does not emerge theoretically but develops gradually through transparency, explainability and positive real-world experience. Future research should focus on long-term, practice-oriented studies that accompany CA implementation in healthcare and evaluate its effects on quality of care, professional workflows, patient safety and the development of clinical expertise. Longitudinal studies are particularly needed to explore the role of early adopters in overcoming the trialability paradox and to develop context-specific implementation strategies for both inpatient and outpatient settings. Overall, this study demonstrates that CAs have the potential to support healthcare delivery, provided their introduction is evidence-based, ethically sound and aligned with professional and legal standards.

## Supplementary Information

Below is the link to the electronic supplementary material.

Supplementary Material 1

Supplementary Material 2

Supplementary Material 3

Supplementary Material 4

Supplementary Material 5

## Data Availability

The datasets generated and/or analysed during the current study are available from the corresponding author upon reasonable request.

## Abbreviations

AI: Artificial intelligence
CA: Conversational agent
CDSS: Clinical decision support systems
COREQ: Consolidated criteria for Reporting Qualitative research

## References

[1] Liebrich F. Digitale medienprodukte in der Arzt-Patienten-Kommunikation: Chancen und Risiken einer personalisierten Medizin. 1. Aufl. 2017 Edition. Wiesbaden: Springer Vieweg. 2017.

[2] Wutz M, Hermes M, Winter V, Köberlein-Neu J. Factors Influencing the Acceptability, Acceptance, and Adoption of Conversational Agents in Health Care: Integrative Review. J Med Internet Res. 2023;25:e46548. 10.2196/46548. 37751279 10.2196/46548 PMC10565637

[3] Laranjo L, Dunn AG, Tong HL, Kocaballi AB, Chen J, Bashir R, et al. Conversational agents in healthcare: a systematic review. J Am Med Inform Assoc. 2018;25:1248–58. 10.1093/jamia/ocy072. 30010941 10.1093/jamia/ocy072 PMC6118869

[4] Laymouna M, Ma Y, Lessard D, Schuster T, Engler K, Lebouché B, Roles. Users, Benefits, and Limitations of Chatbots in Health Care: Rapid Review. J Med Internet Res. 2024;26:e56930. 10.2196/56930. 39042446 10.2196/56930 PMC11303905

[5] Milne-Ives M, de Cock C, Lim E, Shehadeh MH, de Pennington N, Mole G, et al. The Effectiveness of Artificial Intelligence Conversational Agents in Health Care: Systematic Review. J Med Internet Res. 2020;22:e20346. 10.2196/20346. 33090118 10.2196/20346 PMC7644372

[6] McDuff D, Schaekermann M, Tu T, Palepu A, Wang A, Garrison J, et al. Towards accurate differential diagnosis with large language models. Nature. 2025;642:451–7. 10.1038/s41586-025-08869-4. 40205049 10.1038/s41586-025-08869-4 PMC12158753

[7] Palanica A, Flaschner P, Thommandram A, Li M, Fossat Y. Physicians’ Perceptions of Chatbots in Health Care: Cross-Sectional Web-Based Survey. J Med Internet Res. 2019;21:e12887. 10.2196/12887. 30950796 10.2196/12887 PMC6473203

[8] McGreevey JD, Hanson CW, Koppel R. Clinical, Legal, and Ethical Aspects of Artificial Intelligence-Assisted Conversational Agents in Health Care. JAMA. 2020;324:552–3. 10.1001/jama.2020.2724. 32706386 10.1001/jama.2020.2724

[9] Almalki M. Exploring the Influential Factors of Consumers’ Willingness Toward Using COVID-19 Related Chatbots: An Empirical Study. Med Arch. 2021;75:50–5. 10.5455/medarh.2021.75.50-55. 34012200 10.5455/medarh.2021.75.50-55 PMC8116098

[10] Prakash AV, Das S. Intelligent conversational agents in mental healthcare services: A thematic analysis of user perceptions. Pac Asia J Assoc Inform Syst. 2020;12. 10.17705/1pais.12201.

[11] Andersson G, Cuijpers P, Carlbring P, Riper H, Hedman E. Guided Internet-based vs. face-to-face cognitive behavior therapy for psychiatric and somatic disorders: a systematic review and meta-analysis. World Psychiatry. 2014;13:288–95. 10.1002/wps.20151. 25273302 10.1002/wps.20151 PMC4219070

[12] Tudor Car L, Dhinagaran DA, Kyaw BM, Kowatsch T, Joty S, Theng Y-L, et al. Conversational Agents in Health Care: Scoping Review and Conceptual Analysis. J Med Internet Res. 2020;22:e17158. 10.2196/17158. 32763886 10.2196/17158 PMC7442948

[13] Davis FD. User acceptance of information technology: system characteristics, user perceptions and behavioral impacts. Int J Man Mach Stud. 1993;38:475–87. 10.1006/imms.1993.1022.

[14] Tong A, Sainsbury P, Craig J. Consolidated criteria for reporting qualitative research (COREQ): a 32-item checklist for interviews and focus groups. Int J Qual Health Care. 2007;19:349–57. 10.1093/intqhc/mzm042. 17872937 10.1093/intqhc/mzm042

[15] Gale NK, Heath G, Cameron E, Rashid S, Redwood S. Using the framework method for the analysis of qualitative data in multi-disciplinary health research. BMC Med Res Methodol. 2013;13:117. 10.1186/1471-2288-13-117. 24047204 10.1186/1471-2288-13-117 PMC3848812

[16] Ritchie J, Lewis J. Qualitative Research Practice: A Guide for Social Science Students and Researchers. Los Angeles, Calif.: SAGE Publications Inc; 2003.

[17] Morse J, Field P, Field PA. Qualitative research methods for health professionals. 2nd edition. Thousand Oaks: Sage Publications, Inc. 1995.

[18] Saks M, Allsop J. Researching health: qualitative, quantitative and mixed methods. 2019.

[19] Patton MQ. Qualitative evaluation and research methods. Newbury Park, Calif: SAGE Publications Inc. 1990.

[20] Yin RK. Case Study Research: Design and Methods. Los Angeles: Sage; 2013.

[21] Venkatesh V, Thong JYL, Xu X. Consumer acceptance and use of information technology: Extending the unified theory of acceptance and use of technology. MIS Q. 2012;36:157–78. 10.2307/41410412.

[22] Klaffke M. Generationen-Management: Konzepte, Instrumente, Good-Practice-Ansätze. 3., akt. Aufl. 2022 Edition. Wiesbaden Heidelberg: Springer Gabler; 2022.

[23] Green J, Thorogood N. Qualitative methods for health research. 4th edition. Los Angeles: SAGE Publications Ltd. 2018.

[24] Saunders B, Sim J, Kingstone T, Baker S, Waterfield J, Bartlam B, et al. Saturation in qualitative research: exploring its conceptualization and operationalization. Qual Quant. 2018;52:1893–907. 10.1007/s11135-017-0574-8. 29937585 10.1007/s11135-017-0574-8 PMC5993836

[25] Helfferich C. Die Qualität qualitativer Daten. Wiesbaden: VS Verlag für Sozialwissenschaften; 2011. 10.1007/978-3-531-92076-4.

[26] Wutz M, Walter P, Gsell. Dialogsysteme im Krankenhaus. Potenziale von Chatbots. Health&Care Management. 2020. https://www.hcm-magazin.de/potenziale-von-chatbots-271831/. Accessed 27 Sept 2024.

[27] Söling S, Köberlein-Neu J, Müller BS, Dinh TS, Muth C, Pfaff H, et al. From sensitization to adoption? A qualitative study of the implementation of a digitally supported intervention for clinical decision making in polypharmacy. Implement Sci. 2020;15:82. 10.1186/s13012-020-01043-6. 32958010 10.1186/s13012-020-01043-6 PMC7507604

[28] Söling S, Demirer I, Köberlein-Neu J, Hower KI, Müller BS, Pfaff H, et al. Complex implementation mechanisms in primary care: do physicians’ beliefs about the effectiveness of innovation play a mediating role? Applying a realist inquiry and structural equation modeling approach in a formative evaluation study. BMC Prim Care. 2023;24:131. 10.1186/s12875-023-02081-x. 37369994 10.1186/s12875-023-02081-x PMC10294464

[29] Dresing T, Pehl T, Praxisbuch interview. transkription & analyse: Anleitungen und Regelsysteme für qualitativ Forschende. 8. Auflage. Marburg: Eigenverlag. 2018.

[30] Parkinson S, Eatough V, Holmes J, Stapley E, Midgley N. Framework analysis: a worked example of a study exploring young people’s experiences of depression. Qualitative Res Psychol. 2016;13:109–29. 10.1080/14780887.2015.1119228.

[31] Shenton AK. Strategies for ensuring trustworthiness in qualitative research projects. Educ Inform. 2004;22:63–75. 10.3233/EFI-2004-22201.

[32] Bohm D. On Dialogue. London: Routledge; 2013. 10.4324/9780203180372.

[33] Rogers EM. Diffusion of Innovations. New York: Free Press; 1995.

[34] Bibault J-E, Chaix B, Guillemassé A, Cousin S, Escande A, Perrin M, et al. A Chatbot Versus Physicians to Provide Information for Patients With Breast Cancer: Blind, Randomized Controlled Noninferiority Trial. J Med Internet Res. 2019;21:e15787. 10.2196/15787. 31774408 10.2196/15787 PMC6906616

[35] Thirunavukarasu AJ, Hassan R, Mahmood S, Sanghera R, Barzangi K, El Mukashfi M, et al. Trialling a Large Language Model (ChatGPT) in General Practice With the Applied Knowledge Test: Observational Study Demonstrating Opportunities and Limitations in Primary Care. JMIR Med Educ. 2023;9:e46599. 10.2196/46599. 37083633 10.2196/46599 PMC10163403

[36] Hirosawa T, Harada Y, Yokose M, Sakamoto T, Kawamura R, Shimizu T. Diagnostic Accuracy of Differential-Diagnosis Lists Generated by Generative Pretrained Transformer 3 Chatbot for Clinical Vignettes with Common Chief Complaints: A Pilot Study. Int J Environ Res Public Health. 2023;20:3378. 10.3390/ijerph20043378. 36834073 10.3390/ijerph20043378 PMC9967747

[37] Hirosawa T, Kawamura R, Harada Y, Mizuta K, Tokumasu K, Kaji Y, et al. ChatGPT-Generated Differential Diagnosis Lists for Complex Case–Derived Clinical Vignettes: Diagnostic Accuracy Evaluation. JMIR Med Inf. 2023;11:e48808. 10.2196/48808. 10.2196/48808 PMC10594139 37812468

[38] Al-Dabbagh SA, Sulaiman HM, Abdulkarim NA. Workload assessment of medical doctors at primary health care centers in the Duhok governorate. Hum Resour Health. 2022;19:117. 10.1186/s12960-021-00664-2. 35090488 10.1186/s12960-021-00664-2 PMC8796551

[39] von dem Knesebeck O, Koens S, Marx G, Scherer M. Perceptions of time constraints among primary care physicians in Germany. BMC Fam Pract. 2019;20:142. 10.1186/s12875-019-1033-5. 31640573 10.1186/s12875-019-1033-5 PMC6805618

[40] Frank J, Merseburger AS, Landmesser J, Brozat-Essen S, Schramm P, Freimann L, et al. Large Language Modelle zur schnellen Vereinfachung der Eingabe von Qualitätssicherungsdaten: Performance-Test mit Echtdaten am Beispiel der Tumordokumentation in der Urologie. Aktuelle Urol. 2024. 10.1055/a-2281-8015 10.1055/a-2281-8015 38599593

[41] Balaji D, He L, Giani S, Bosse T, Wiers R, de Bruijn G-J. Effectiveness and acceptability of conversational agents for sexual health promotion: a systematic review and meta-analysis. Sex Health. 2022;19:391–405. 10.1071/SH22016. 35863761 10.1071/SH22016 PMC7613710

[42] Meyer M, Johnson JD, Ethington C. Contrasting Attributes of Preventive Health Innovations. J Communication. 1997;47:112–31. 10.1111/j.1460-2466.1997.tb02709.x.

[43] Jussupow E, Spohrer K, Heinzl A, Gawlitza J. Augmenting Medical Diagnosis Decisions? An Investigation into Physicians’ Decision-Making Process with Artificial Intelligence. Inform Syst Res. 2021;32:713–35. 10.1287/isre.2020.0980.

[44] Böcken J, Braun B, Meierjürgen R, Gesundheitsmonitor. 2016: Bürgerorientierung im Gesundheitswesen. Verlag Bertelsmann Stiftung; 2016.

[45] Fang ML, Siden E, Korol A, Demestihas M-A, Sixsmith J, Sixsmith A. A Scoping review exploration of the intended and unintended consequences of ehealth on older people: a health equity impact assessment. Human Technol Interdiscipl J Humans ICT Environ. 2018;14:297–323. 10.17011/ht/urn.201811224835.

[46] Nadarzynski T, Bayley J, Llewellyn C, Kidsley S, Graham CA. Acceptability of artificial intelligence (AI)-enabled chatbots, video consultations and live webchats as online platforms for sexual health advice. BMJ Sex Reprod Health. 2020;46:210–7. 10.1136/bmjsrh-2018-200271. 31964779 10.1136/bmjsrh-2018-200271

[47] Damschroder LJ, Reardon CM, Widerquist MAO, Lowery J. The updated Consolidated Framework for Implementation Research based on user feedback. Implement Sci. 2022;17:75. 10.1186/s13012-022-01245-0. 36309746 10.1186/s13012-022-01245-0 PMC9617234

[48] Greenhalgh T, Maylor H, Shaw S, Wherton J, Papoutsi C, Betton V, et al. The NASSS-CAT Tools for Understanding, Guiding, Monitoring, and Researching Technology Implementation Projects in Health and Social Care: Protocol for an Evaluation Study in Real-World Settings. JMIR Res Protoc. 2020;9:e16861. 10.2196/16861. 32401224 10.2196/16861 PMC7254278

[49] Araujo T. Living up to the chatbot hype: The influence of anthropomorphic design cues and communicative agency framing on conversational agent and company perceptions. Comput Hum Behav. 2018;85:183–9. 10.1016/j.chb.2018.03.051.

[50] Abd-Alrazaq AA, Alajlani M, Ali N, Denecke K, Bewick BM, Househ M. Perceptions and Opinions of Patients About Mental Health Chatbots: Scoping Review. J Med Internet Res. 2021;23:e17828. 10.2196/17828. 33439133 10.2196/17828 PMC7840290

[51] Denis J-L, Hébert Y, Langley A, Lozeau D, Trottier L-H. Explaining diffusion patterns for complex health care innovations. Health Care Manage Rev. 2002;27:60–73. 10.1097/00004010-200207000-00007. 12146784 10.1097/00004010-200207000-00007

[52] Grimshaw JM, Thomas RE, MacLennan G, Fraser C, Ramsay CR, Vale L, et al. Effectiveness and efficiency of guideline dissemination and implementation strategies. Health Technol Assess. 2004;8:iii–iv. 10.3310/hta8060. 14960256 10.3310/hta8060

[53] Davis FD, Perceived, Usefulness. Perceived Ease of Use, and User Acceptance of Information Technology. MIS Q. 1989;13:319–40. 10.2307/249008.

[54] Heerink M, Kröse B, Evers V, Wielinga B. Assessing Acceptance of Assistive Social Agent Technology by Older Adults: the Almere Model. Int J Soc Rob. 2010;2:361–75. 10.1007/s12369-010-0068-5.

[55] Greenhalgh T, Robert G, Macfarlane F, Bate P, Kyriakidou O. Diffusion of Innovations in Service Organizations: Systematic Review and Recommendations. Milbank Q. 2004;82:581–629. 10.1111/j.0887-378X.2004.00325.x. 15595944 10.1111/j.0887-378X.2004.00325.x PMC2690184

[56] Kennedy CM, Powell J, Payne TH, Ainsworth J, Boyd A, Buchan I. Active assistance technology for health-related behavior change: an interdisciplinary review. J Med Internet Res. 2012;14:e80. 10.2196/jmir.1893. 22698679 10.2196/jmir.1893 PMC3415065

[57] ter Stal S, Kramer LL, Tabak M, op den Akker H, Hermens H. Design Features of Embodied Conversational Agents in eHealth: a Literature Review. Int J Hum Comput Stud. 2020;138:102409. 10.1016/j.ijhcs.2020.102409.

[58] Lehne M, Sass J, Essenwanger A, Schepers J, Thun S. Why digital medicine depends on interoperability. NPJ Digit Med. 2019;2:79. 10.1038/s41746-019-0158-1. 31453374 10.1038/s41746-019-0158-1 PMC6702215

