# Supplementary material for: Physicians’ expectations of the use of conversational agents in healthcare: a qualitative study
Source: BMC Health Serv Res. 2026 Mar 12;26:485. doi: 10.1186/s12913-026-14321-8 (PMC13063729; doi:10.1186/s12913-026-14321-8)
Supplement: Supplementary file 1 — Supplementary Material 1 [file 12913_2026_14321_MOESM1_ESM.pdf]

Additional file 1: Pre-questionnaire

“Physicians' expectations of the use of conversational agents in healthcare”

Sehr geehrte Dame, Sehr geehrter Herr,

vielen Dank, dass Sie sich als Interviewpartner:in für unsere Studie zur Wahrnehmung von Ärzt:innen über den Einsatz von Conversational Agents (CA) im Gesundheitswesen zur Verfügung stellen. Bitte füllen Sie diesen kurzen Fragebogen aus und senden diesen anschließend an maximilian.wutz@uni-wuppertal.de. Vielen Dank.

1 Name:

2 Alter/Jahrgang:

3 Geschlecht:

4 Berufsbezeichnung:

Fragesupport: *Bitte geben Sie an, ob Sie eine eigene Praxis haben oder ob Sie in einem Klinikum angestellt sind bzw. arbeiten. Bitte geben Sie darüber hinaus Ihre genaue Berufsbezeichnung (Fachärztin/-arzt, Assistenzärztin/-arzt etc.) an.*

☐ Ärztin/Arzt mit eigener Praxis ☐ Angestellte Ärztin/Arzt in einer Klinik

Fachärztin/-arzt für:

5 Führungstätigkeit:

Fragesupport: *Bitte geben Sie an, ob Sie in Ihrer derzeitigen Anstellung als Führungskraft tätig sind.*

☐ Ja ☐ Nein

6 Erfahrungen mit Conversational Agents:

Fragesupport: *Bitte geben Sie an, ob Sie bereits Erfahrungen mit Conversational Agents gemacht haben. Falls ja, geben Sie bitte darüber hinaus auch die jeweiligen Bereiche (Gesundheitswesen, Kundenservice etc.) an.*

☐ Ja ☐ Nein

In welchen Bereichen?

7 Technikaffinität:

Fragesupport: *Bitte geben Sie an, ob Sie sich als technikaffin bezeichnen würden.*

☐ Ja ☐ Nein

8 Wünschen Sie die Übersendung des Leitfadens für das geplante Interview vor dem geplanten Interviewtermin?

☐ Ja ☐ Nein

9 Bevorzugter Interviewtermin:

Fragesupport: *Bitte teilen Sie uns Ihren bevorzugten Interviewtermin oder Zeitraum zwischen dem 02.11.2023 und dem 31.01.2024 für das Interview mit. Gerne können Sie uns auch mehrere Daten oder Zeiträume nennen. Wir werden versuchen diese zu berücksichtigen.*

10 Bevorzugtes Interviewtool:

Fragesupport: *Bitte teilen Sie uns Ihr bevorzugtes Tool für die Durchführung des Interviews mit. Sollten Sie das Interview in Präsenz führen wollen, geben Sie dies bitte an. Wir werden versuchen alle Wünsche zu berücksichtigen.*

☐ Teams ☐ Zoom ☐ Skype

Anderes:

11 E-Mail-Adresse:

Fragesupport: *Bitte teilen Sie uns zum Abschluss noch Ihre E-Mail-Adresse mit, sodass wir mit Ihnen bezüglich eines Interviewtermins in Kontakt treten können. Sollten Sie andere Kommunikationsart bevorzugen, geben Sie diese bitte an (bspw. Telefonnummer).*

Vielen Dank für Ihre Bemühungen. Ihre Angaben und Daten verwenden wir selbstverständlich absolut vertraulich und anonymisiert. Bezüglich Terminierung des Interviews werden wir uns in Kürze bei Ihnen melden. Hierbei werden wir versuchen Ihren bevorzugten Interviewtermin einzuhalten.

English version:

“Physicians' expectations of the use of conversational agents in healthcare”

Dear Ladies and Gentlemen,

thank you for making yourself available as an interview partner for our study on physicians' expectations of the use of conversational agents (CA) in healthcare. Please complete this short questionnaire and then send it to maximilian.wutz@uni-wuppertal.de. Thank you very much.

1 Name:

☐

2 Age/year of birth:

☐

3 Gender:

☐

4 Job title:

Question support: Please state whether you have your own practice or whether you are employed or work in a hospital. Please also state your exact job title (specialist physician, assistant physician, etc.).

☐ Physician with own practice ☐ Employed physician in a clinic

Specialist physician for: ☐

5 Leadership activity:

Question support: Please indicate whether you are a manager in your current job.

☐ Yes ☐ No

6 Experience with Conversational Agents:

Question support: Please indicate whether you have already had experience with conversational agents. If yes, please also indicate the relevant areas (healthcare, customer service, etc.).

☐ Yes ☐ No

In which areas? ☐

7 Technical affinity:

Question support: Please indicate whether you would describe yourself as tech-savvy.

☐ Yes ☐ No

8 Would you like to receive the guidelines for the planned interview before the scheduled interview date?

☐ Yes ☐ No

9 Preferred interview date:

Question support: Please let us know your preferred interview date or period between 02.11.2023 and 31.01.2024 for the interview. You are also welcome to give us several dates or periods. We will try to take these into account.

10 Preferred interview tool:

Question support: Please let us know your preferred tool for conducting the interview. If you would like to conduct the interview in person, please indicate this. We will try to accommodate all requests.

☐ Teams ☐ Zoom ☐ Skype

Other:

11 E-Mail-address:

Question support: Finally, please let us know your e-mail address so that we can get in touch with you regarding an interview appointment. If you prefer another method of communication, please indicate this (e.g. telephone number).

Thank you for your efforts. We will of course use your details and data in absolute confidence and anonymously. We will contact you shortly regarding the scheduling of the interview. We will try to keep to your preferred interview date.
