# Supplementary material for: Physicians’ expectations of the use of conversational agents in healthcare: a qualitative study
Source: BMC Health Serv Res. 2026 Mar 12;26:485. doi: 10.1186/s12913-026-14321-8 (PMC13063729; doi:10.1186/s12913-026-14321-8)
Supplement: Supplementary file 2 — Supplementary Material 2 [file 12913_2026_14321_MOESM2_ESM.pdf]

Additional file 2: Interview Guide

Interviewleitfaden zur Studie “Physicians' expectations of the use of conversational agents in healthcare”

Einleitungs- beziehungsweise Vorstellungsphase

1. Dank für die Gesprächsbereitschaft
2. Einverständnis für Protokollierung
3. Anonymisierung der Antworten erläutern
4. Vorstellung des Interviewers
5. Interviewzeitrahmen benennen (circa 20-45 Minuten)
6. Erläuterung des Themas der Untersuchung

Nr.	Fragestellung	Nachfragen
Frage 1 (CA-Erfahrung)	In der Vorabfrage gaben Sie an, dass Sie bereits schon einmal etwas von CAs gehört haben und bereits Kontakt beziehungsweise Erfahrungen mit dieser Technologie hatten. Können Sie mir davon kurz berichten?	In welchem Zusammenhang haben Sie von CAs gehört?<br><br>In welchem Bereich hatten Sie bereits Kontakt mit CAs?
Frage 1 (ohne CA-Erfahrung)	In der Vorabfrage gaben Sie an, dass Sie bisher noch nichts von CAs gehört haben und noch keinen Kontakt beziehungsweise Erfahrung mit dieser Technologie hatten. Ist dies korrekt? Was sind Ihrer Meinung nach die Hintergründe hierfür?	
Frage 2	Wie denken Sie über den Einsatz von CAs im Gesundheitswesen und wie ist Ihre Wahrnehmung zu dieser Technologie?	In welchen Bereichen des Gesundheitswesens sehen Sie den Einsatz von CAs? (konkrete Anwendungsbeispiele)<br><br>Können Sie sich vorstellen CAs in Ihrer täglichen Arbeit zu nutzen und diese Patient:innen zur Versorgung zu verschreiben? (konkretes Anwendungsbeispiel)<br><br>In welcher Form sehen Sie die Zusammenarbeit/den Einsatz von CAs (unterstützend oder ersetzend)?
Frage 3	Stellen Sie sich vor Sie könnten sich frei nach Ihrem Wunsch einen Gesundheits-CA erstellen.	Rückfragen zu einzelnen (genannten und nicht genannten, aber in der Literatur bestätigten) Faktoren

	3.1: Wie müsste der CA beschaffen sein bzw. welche Eigenschaften muss der CA besitzen, dass die Technologie von Ihnen/Ärzt:innen in der Patientenversorgung und allgemein im täglichen Arbeitsprozess genutzt wird? (Erläuterung gerne anhand eines Beispiels).<br><br>3.2: Wie müsste der CA beschaffen sein, dass Sie/Ärzt:innen die Technologie verschreiben? (Erläuterung gerne anhand eines Beispiels).<br><br>3.3. Welche Eigenschaften muss der CA besitzen, dass dieser von den Patient:innen genutzt wird? (Erläuterung gerne anhand eines Beispiels)	
Frage 4	Stellen Sie sich einmal vor, sie würden CAs in Ihrem Versorgungsalltag anwenden wollen, was bräuchte es um Sie herum, um Sie darin zu unterstützen (Rahmenbedingungen/Gesetze)? Worin sehen Sie derzeit die größten Herausforderungen?	Bitte verwenden Sie ein konkretes Beispiel aus Ihrem Versorgungsalltag, um dies zu beschreiben.
Frage 5	Potentiale und Risiken<br><br>5.1: Was sind aus Ihrer Sicht die relevanten Potentiale und Risiken von CAs für ihre tägliche Arbeit? (Erläuterung gerne anhand eines Beispiels)<br><br>5.2: Was sind aus Ihrer Sicht die relevanten Potentiale und Risiken von CAs in der Versorgung von Patient:innen? (Erläuterung gerne anhand eines Beispiels)<br><br>5.3: Was muss passieren, dass die Potentiale von CAs genutzt werden?<br><br>5.4: Was muss passieren, dass die genannten Risiken beseitigt werden?	Rückfragen zu einzelnen (genannten und nicht genannten, aber in der Literatur bestätigten) Faktoren

Rückblick

1. Kurze Zusammenfassung des Gesagten
   Rückfrage: Haben Sie den Eindruck, dass noch Punkte, die aus ihrer Sicht für die Untersuchung relevant sind, vergessen wurden?
2. Erneuter Dank für die Zeit

Ausblick

1. Information über Auswertung der Ergebnisse
2. Übersendung der Ergebnisse nach Veröffentlichung gewünscht?

English version:

Interview guide for the study "Physicians' expectations of the use of conversational agents in healthcare"

Introductory and presentation phase:

1. Thanks for the willingness to talk
2. Consent for recording
3. Explain anonymization of the answers
4. Introduction of the interviewer
5. Interview time frame (approx. 20-45 minutes)
6. Explanation of the topic of the study

Number	Question	Follow-up questions
Question 1 (CA experience)	In the preliminary question, you stated that you had already heard of CAs and had already had contact or experience with this technology. Can you briefly tell me about this?	In what context have you heard of CAs?<br><br>In which area have you already had contact with CAs?
Question 1 (without CA experience)	In the preliminary question, you stated that you had not yet heard of CAs and had not yet had any contact or experience with this technology. Is this correct? In your opinion, what are the reasons for this?	
Question 2	What do you think about the use of CAs in healthcare and what is your perception of this technology?	In which areas of healthcare do you see the use of CAs? (specific application examples)<br><br>Can you imagine using CAs in your daily work and prescribing them to patients for their care? (concrete application example)<br><br>In what form do you see the collaboration/use of CAs (supporting or replacing)?
Question 3	Imagine you could create a healthcare CA as you wish.<br><br>3.1: How would the CA have to be designed or what properties must the CA have so that the technology is used by you/physicians in patient care and generally in the daily work process? (Please explain using an example).<br><br>3.2: How should the CA be designed so that you/physicians prescribe the technology? (Please explain with an example).	Queries on individual factors (mentioned and not mentioned, but confirmed in the literature)

	3.3: What characteristics must the CA have for it to be used by patients? (Please explain with an example)	
Question 4	Imagine you wanted to use CAs in your day-to-day care, what would it take around you to support you in this (framework conditions/laws)? What do you currently see as the biggest challenges?	Please use a concrete example from your everyday care routine to describe this.
Question 5	Potentials and risks<br><br>5.1: In your opinion, what are the relevant potentials and risks of CAs for your daily work? (Please explain using an example)<br><br>5.2: In your opinion, what are the relevant potentials and risks of CAs in the care of patients? (Please explain with an example)<br><br>5.3: What needs to happen for the potential of CAs to be utilized?<br><br>5.4: What needs to happen to eliminate the risks mentioned?	Queries on individual factors (mentioned and not mentioned, but confirmed in the literature)

Review:

1. Brief summary of what has been said
   Query: Do you have the impression that points relevant to the investigation from your point of view have been forgotten?
2. Thanks again for participation.

Outlook:
1. Information on the evaluation of the results
2. Would you like to receive the results after publication?
