# Supplementary material for: Physicians’ expectations of the use of conversational agents in healthcare: a qualitative study
Source: BMC Health Serv Res. 2026 Mar 12;26:485. doi: 10.1186/s12913-026-14321-8 (PMC13063729; doi:10.1186/s12913-026-14321-8)
Supplement: Supplementary file 4 — Supplementary Material 4 [file 12913_2026_14321_MOESM4_ESM.pdf]

Additional file 4: Original quotes in German and the English translation

Interview partner (IP)	Original quote (German)	English translation
IP-8	„Das Gesundheitswesen ist ein budgetiertes System und für Innovationen fehlt uns ganz klar das Budget. Aber natürlich hat es auch mit Menschen zu tun. Und mit den Bedingungen wie Regulationen, Datenschutz und Ängsten, dass man was falsch machen könnte. […] Das hat auch weitere verschiedene Gründe, vor allen Dingen auch die, dass wir chronisch unterbesetzt sind und keine Zeit habe neue Technologien auszuprobieren und einzuführen."	"Healthcare is a budget-driven system, and we clearly lack the budget for innovations. But of course, it also has to do with people. And with conditions such as regulations, data protection and fears that you might do something wrong. [...] There are also various other reasons, above all the fact that we are chronically understaffed and have no time to try out and implement new technologies."
IP-9	„Ich glaube, das ist einfach noch zu zukunftsorientiert. So modern sind die Krankenhäuser größtenteils noch nicht. Wenn sie noch nicht mal digital sind, dann glaube ich, kann man CAs und AI noch nicht integrieren.“	"I think it's just too future-oriented. Most hospitals are not that modern yet. If they're not even digital, then I don't think you can integrate CAs and AI yet."
IP-10	„Die Verknüpfung mit den bestehenden Systemen. Der CA muss auf verschiedene Systeme anpassbar sein und sich einfach in die bestehenden Systeme einfügen.	"The integration into existing systems. The CA must be adaptable to different systems and easy to integrate into existing systems."
IP-6	„Ich glaube da ist jetzt der Gesetzgeber stark gefragt, um Lösungen zu entwickeln und sich auch mal was zu trauen.“	"I think it is now up to legislators to develop solutions and have the courage to do something."
IP-3	„Das Thema Datenschutz wird oft bei neuen Innovationen als Grund vorgeschoben, aber das lässt sich ehrlichweise alles lösen.“	"The issue of data protection is often put forward as a reason for new innovations, but it can honestly all be solved."
IP-5	„CAs werden zukünftig eine immer größere Rolle im Gesundheitswesen spielen und fester Bestandteil der Gesundheitsversorgung sein, weil wir Ärzte immer mehr Patienten versorgen müssen und dann werden wir die Unterstützung solcher Chatbots benötigen.“	"CAs will play an increasingly important role in healthcare in the future and will be an integral part of healthcare provision, because we physicians will have to care for more and more patients, and then we will need the support of such chatbots."
IP-16	„Unterstützung erhoffe ich mir vor allem in der Dokumentation. Ich habe das Gefühl, wenn ich nicht so viel dokumentieren müsste, wie ich es tue, dann würde ich	"I'm hoping for the greatest support with documentation. I have the feeling that I could treat three times as many patients if I didn't have to document as much as I do."

	dreimal so viele Patienten behandeln können.“	
IP-9	„[…] man erhofft sich auch einen schnelleren Zugang zur Versorgung und auch den richtigen Zugang. Also, dass der Patient direkt vorgeschlagen bekommt, das sind die richtigen Fachkräfte für die Beschwerden und nicht, dass alle in die Notaufnahme gehen, sondern direkt in den richten Versorgungsbereich […].“	“[...] the hope is also faster access to care and the appropriate access. In other words, the patient is told directly that these are the right specialists for their complaints, and not everyone is sent to the emergency room, but sent directly to the right care area [...].”
IP-15	„[...] Routine- und einfache Aufgaben können schon heute von CAs übernommen werden, um dem Fachkräftemangel entgegenzuwirken.“	“[…] routine and simple tasks will be performed by CAs in order to counteract the shortage of specialists.”
IP-2	„CAs können die Effizienz von Behandlung und Diagnostik erheblich steigern, da die CA auch nicht offensichtliche und seltene Krankheiten berücksichtigen, Anamnese, Laborwerte und Vorgeschichte des Patienten in kurzer Zeit zusammenfassen, Medikamentenunverträglichkeiten erkennen und Vergleichsfälle mit ähnlichem Verlauf abrufen kann."	“CAs can significantly increase the efficiency of treatment and diagnostics, as the CA can also take into account non-obvious and rare diseases, summarize the patient’s medical history, laboratory values and past history in a short time, identify drug intolerances and call up comparative cases with a similar course”
IP-16	„Solange CAs ein extra System sind, glaube ich, haben wir keinen Qualitätsverlust. Sobald sie aber das medizinische Personal ersetzen, wird es automatisch zu einem Qualitätsverlust führen.“	“As long as CAs are an extra system, I don't think we will have a loss of quality. But as soon as they replace the medical staff, it will automatically lead to a loss of quality.”
IP-5	„Die gesamte Versorgung und unsere tagtägliche Arbeit wird sich durch den Einsatz von Technologie verändern. Damit wir mit den Systemen auch umgehen können und verstehen wie sie funktionieren brauchen wir auch eine IT-lastigere Ausbildung bei medizinischen Berufen.“	“The entire healthcare system and our day-to-day work will change through the use of technology. In order for us to be able to handle the systems and understand how they work, we also need more IT-oriented training for medical professions.”
IP-5	„Also wir werden ja immer mehr unsere Patienten auch im häuslichen Bereich behandeln, da es immer mehr ältere Patienten geben und zu immer mehr unversorgten Gebieten kommen wird. Das heißt die Patienten sind zu Hause und sollen überwacht werden. Dies kann auch durch eine Technologie wie CAs	“We will be treating more and more of our patients at home, as there will be more and more elderly patients and more and more untreated areas. This means that patients are at home and should be monitored. This can also be done using technology such as CAs, which collects the

	erfolgen, die die Informationen gesammelt und strukturiert an mich weiterleitet.“	information and forwards it to me in a structured way.”
IP-5	„Die CA muss ein dauerhaft funktionierendes System sein, das praktisch keine technischen Ausfälle aufweist und stets seinen Zweck erfüllt. Ist dies nicht der Fall, würde ich es nicht verwenden und meinen Patienten auch nicht empfehlen.“	“The CA must be a permanently functioning system that has practically no technical failures and always fulfils its purpose. If it doesn’t, I wouldn’t use it and wouldn’t recommend it to my patients.”
IP-8	„benutzerfreundlich und für jeden bedienbar“	“user-friendly and operable by everyone”
IP-12	„jeder hat einen anderen Geschmack, Vorlieben und Bedürfnisset.“	“everyone has different tastes, preferences and needs”
IP-6	„zuverlässig sein, d.h. korrekte Diagnosen stellen, die bestmögliche Behandlung vorschlagen und seine Informationen aus einer glaubwürdigen und evidenzbasierten Quelle haben. Gleichzeitig will ich auch nicht immer alles hinterfragen müssen, sondern ein Sicherheitsgefühl bekommen.“	“be reliable, i.e. make correct diagnoses, suggest the best possible treatment and have information from a credible and evidence-based source. At the same time, I don't always want to have to question everything; I want to have a sense of security”
IP-4	„Auch hier kommt es zu einer klassischen Kosten-Nutzen-Analyse. Die Kosten für Einführung und Nutzen dürfen aber nicht zu hoch sein, sonst wird es natürlich wegen unserem limitierten Budget nicht eingeführt.“	“A classic cost–benefit analysis is also carried out here. However, the costs of introduction and benefits must not be too high, otherwise it will of course not be introduced due to our limited budget.”
