# Supplementary material for: Physicians’ expectations of the use of conversational agents in healthcare: a qualitative study
Source: BMC Health Serv Res. 2026 Mar 12;26:485. doi: 10.1186/s12913-026-14321-8 (PMC13063729; doi:10.1186/s12913-026-14321-8)
Supplement: Supplementary file 5 — Supplementary Material 5 [file 12913_2026_14321_MOESM5_ESM.pdf]

Additional file 5: Risks mentioned by the physicians interviewed

This table summarizes the potentials and risks of CAs. For a clearer presentation of the potentials and risks in the table, different shades of gray are used depending on the target group (Potentials and risks for physicians and patients are shaded in white, those that only affect physicians are shaded in light gray, and those that only affect patients are shaded in dark gray).

Potentials	Risks
Relief for the healthcare system	More time-intensive treatments
Relief for healthcare professionals	Over-diagnostics
Increased quality of care	Patient safety
Individualized treatment	Loss of quality with replacing use
Easier and faster access to care	Unlearning the medical profession, too much reliance on the CA
Combating the shortage of skilled workers	Loss of confidence in one's own medical abilities
Better supply in rural areas	Legal issues (liability, responsibility for errors)
Making work easier for physicians	Data privacy
Sense of security for physicians	Lack of adoption
Broad knowledge among physicians	Misdiagnoses
Cost savings	Increased costs
Time saving	
Permanent availability	
Speeding up and simplifying processes	
Non-judgmental property	
Fewer unnecessary visits to the physician	
